# AI-Driven Cell Tracking to Enable High-Throughput Drug Screening Targeting Airway Epithelial Repair for Children with Asthma

**DOI:** 10.3390/jpm12050809

**Published:** 2022-05-17

**Authors:** Alphons Gwatimba, Tim Rosenow, Stephen M. Stick, Anthony Kicic, Thomas Iosifidis, Yuliya V. Karpievitch

**Affiliations:** 1 Wal-Yan Respiratory Research Centre, Telethon Kids Institute, University of Western Australia, Perth, WA 6009, Australia; tim.rosenow@uwa.edu.au (T.R.); stephen.stick@telethonkids.org.au (S.M.S.); anthony.kicic@telethonkids.org.au (A.K.); thomas.iosifidis@telethonkids.org.au (T.I.); yuliya.karpievitch@telethonkids.org.au (Y.V.K.); 2 School of Computer Science and Software Engineering, University of Western Australia, Nedlands, WA 6009, Australia; 3 Centre for Microscopy, Characterisation and Analysis, University of Western Australia, Nedlands, WA 6009, Australia; 4 Division of Paediatrics, Medical School, University of Western Australia, Nedlands, WA 6009, Australia; 5 Department of Respiratory and Sleep Medicine, Perth Children’s Hospital, Nedlands, WA 6009, Australia; 6 Centre for Cell Therapy and Regenerative Medicine, School of Medicine, University of Western Australia, Nedlands, WA 6009, Australia; 7 School of Population Health, Curtin University, Bentley, WA 6102, Australia; 8 School of Biomedical Sciences, University of Western Australia, Nedlands, WA 6009, Australia

**Keywords:** asthma, wound repair, artificial intelligence, deep learning, cell tracking, cell detection, cell migration, bioinformatics, image analysis, computational biology

## Abstract

The airway epithelium of children with asthma is characterized by aberrant repair that may be therapeutically modifiable. The development of epithelial-targeting therapeutics that enhance airway repair could provide a novel treatment avenue for childhood asthma. Drug discovery efforts utilizing high-throughput live cell imaging of patient-derived airway epithelial culture-based wound repair assays can be used to identify compounds that modulate airway repair in childhood asthma. Manual cell tracking has been used to determine cell trajectories and wound closure rates, but is time consuming, subject to bias, and infeasible for high-throughput experiments. We therefore developed software, EPIC, that automatically tracks low-resolution low-framerate cells using artificial intelligence, analyzes high-throughput drug screening experiments and produces multiple wound repair metrics and publication-ready figures. Additionally, unlike available cell trackers that perform cell segmentation, EPIC tracks cells using bounding boxes and thus has simpler and faster training data generation requirements for researchers working with other cell types. EPIC outperformed publicly available software in our wound repair datasets by achieving human-level cell tracking accuracy in a fraction of the time. We also showed that EPIC is not limited to airway epithelial repair for children with asthma but can be applied in other cellular contexts by outperforming the same software in the Cell Tracking with Mitosis Detection Challenge (CTMC) dataset. The CTMC is the only established cell tracking benchmark dataset that is designed for cell trackers utilizing bounding boxes. We expect our open-source and easy-to-use software to enable high-throughput drug screening targeting airway epithelial repair for children with asthma.

## 1. Introduction

Frequent exposure to pathogens, allergens and pollutants results in damage of the epithelial cell layer lining the airways [1]. Following an injury resulting in the disruption of the epithelium, cells at the wound site, referred to as leading edge cells, migrate to heal the injured epithelium and restore the physical cellular barrier [1,2]. However, in patients with asthma, airway epithelial cells fail to restore epithelial integrity following injury, resulting in further damage and inflammation. Furthermore, dysregulated epithelial repair in children even with mild asthma may contribute to the persistence of asthma into adulthood [3]. Accordingly, the identification of therapeutic treatments that restore the repair properties of wounded epithelial cells by screening and assessing the efficacy of different novel or repurposed drugs is essential. High-throughput drug discovery and repurposing experiments focusing on enhancing airway wound healing will constitute a precision-medicine approach to asthma treatment. Such an approach will target the underlying disease processes and reduce disease burden at the early stages of childhood asthma.

Various in vitro assays can be used to study wound repair mechanisms [4] and screen therapeutics that modulate cell migration and repair [5]. The in vitro scratch assay is widely used to assess wound repair outcomes and is scalable for high-throughput screening purposes [6,7,8]. While the method is primarily used to quantitatively assess the migration characteristics of cell populations, it has been extended to include the analysis of individual cell trajectories using time lapse live cell microscopy [5,9,10]. Cell trajectories are currently obtained by manually tracking a number of leading edge cells (commonly 20) across multiple time lapse image frames generated using the extended in vitro scratch assay [5]. Cell migration metrics, such as velocity and directionality, are then quantified from the cell trajectories and used to assess wound repair outcomes, such as the acceleration or delaying of cell migration for wound healing, due to an administered drug.

Manual cell tracking is laborious, slow, subject to bias [11,12] and cannot be feasibly used to analyze the large volumes of data generated in high-throughput drug screening experiments. While automated cell tracking solutions exist [13,14,15], many require cells to be fluorescently labelled [10,16,17], which can cause undesirable changes to cell behavior or even result in cell death [17]. Many solutions are also not truly fully automated, which is infeasible for high-throughput experiments. For example, fully automated tracking without first manually selecting the cells to track is presented as a convenience primarily for fluorescent cells in [11] and suggested mainly for relatively easy use cases in [18]. Additionally, some solutions do not publicly release the software [19,20] and most do not include the capacity to automatically perform useful wound repair analyses, such as automatic cell migration metric quantification [19,21,22,23,24]. In contrast, if this capacity is included, the solution is hindered by one or more of the previously mentioned limitations [25].

Automated cell trackers generally operate by first outlining the exact shapes of, or segmenting, cells in images before tracking their movements across multiple frames [15]. Cell segmentation is commonly performed using traditional segmentation algorithms [26], such as intensity thresholding [17,27]. However, such methods often struggle to resolve individual cells [16], especially in images with high cell density [28] such as wound repair images. Additionally, optimal segmentation parameter selection is difficult and time consuming [29,30]. Instead, artificial intelligence (AI), or more precisely deep learning (DL)-based, segmentation methods [31], which are increasingly utilized by many cell trackers, better identify individual cells and require minimal runtime parameter finetuning [16,24,32,33]. However, to achieve such performance these systems must be trained using images containing many cells that have been manually segmented. Specifically, to obtain the training data, the exact outline of hundreds of cells must be carefully drawn in multiple images (Supplementary Figure S1, Additional File S1), which is time consuming and often challenging for users [34,35]. Alternatively, AI-based methods that do not rely on stringent object segmentation but instead use rectangular bounding boxes to enclose objects for detection [36] require training data that are easier and faster to generate [34,35]. Specifically, obtaining the training data only requires two clicks of a mouse to specify the upper-leftmost and lower-rightmost corners of a bounding box for each cell in the dataset (Supplementary Figure S1, Additional File S1). Most mainstream object trackers use bounding boxes to detect objects [37,38,39], such as pedestrians and cars. For example, ByteTrack [40] accurately tracks detected objects by first associating high score bounding boxes across frames followed by low score ones. TransMOT [41] is a computationally efficient tracker that models the spatial temporal relationships of objects detected using bounding boxes. On the other hand, cell trackers are overwhelmingly segmentation dependent, thereby restricting end users. For example, while a recent cell tracking algorithm (CellTrack R-CNN) could generate and utilize bounding boxes, a trained segmentation model (Mask R-CNN [42]) was still required to track cells [43].

Importantly, most automated trackers rely on algorithms that do not presuppose that image sequences have low resolution (contain objects smaller than 32 by 32 px [44]) and low framerate (have longer than 25 min frame intervals [19]). With a higher framerate, objects are imaged very frequently across time and are displaced by only a few pixels in adjacent frames and are thus easier to track [45,46,47]. Whereas with a lower framerate, the movements of cells, especially those exhibiting drug-accelerated migration, are often larger than distances to nearby cells, thus making tracking harder [19]. In addition, low-resolution cells are harder to detect [44]. Existing solutions do not target challenging use cases with both low resolution and low framerate [22,23,48,49,50]. Hence, existing solutions do not guarantee reliable cell tracking accuracy.

Here, we present EPIC, a fully automated cell tracking software solution that overcomes all the previously mentioned limitations (EPIC is available at: https://github.com/AlphonsG/EPIC-BBox-Cell-Tracking, accessed on 9 February 2022). EPIC uses state-of-the-art AI-based Vision Transformers [51], which we have previously applied to cell image analysis tasks [52], to accurately detect unstained cells using bounding boxes for decreased labor and time necessary for training data generation compared to widely used segmentation-based approaches. We developed a custom object tracking algorithm for high accuracy tracking in low-resolution and low-framerate image sequences. After completing cell tracking, EPIC automatically generates reports with several cell migration metrics and publication-ready figures. We evaluated EPIC using an airway epithelial cell wound repair dataset generated under high-throughput drug screening experimental conditions. EPIC produced cell migration metrics that were comparable to the current gold standard method, manual cell tracking, and outperformed publicly available automated trackers tested on the same dataset. We also showed that EPIC is not limited to airway epithelial repair for children with asthma but can be applied in other cellular contexts. Specifically, EPIC also outperformed the same automated trackers on the recent Cell Tracking with Mitosis Detection Challenge (CTMC) dataset [36], the only established cell tracking benchmark dataset, which is challenging and diverse with 14 different cell lines, that is designed for cell trackers utilizing bounding boxes. We expect our open-source and easy-to-use software to enable high-throughput drug screening targeting airway epithelial repair for children with asthma.

## 2. Materials and Methods

### 2.1. Datasets

#### 2.1.1. Wound Repair Dataset

We generated a wound repair dataset under high-throughput drug screening experimental conditions. We obtained human telomerase reverse transcriptase modified airway epithelial cells (NuLi-1) [53] from the American Type Culture Collection (ATCC, Manassas, VA, USA) and cultured cells as previously described [54] using bronchial epithelial basal medium (BEBM™, Lonza, Basel, Switzerland) supplemented with SingleQuot growth additives (Lonza). We utilized an established in vitro scratch assay to assess epithelial cell repair responses to wounding as previously described [5]. Briefly, we established monolayer cell cultures in IncuCyte^®^ ImageLock 96-well plates (Essen Bioscience Inc., Ann Arbor, WI, USA) in culture media lacking epidermal growth factor. We wounded confluent monolayer cultures using the IncuCyte^®^ 96-well WoundMaker Tool (Essen Bioscience) [5]. We then treated subsets of cell cultures with either a specific Akt inhibitor (10 µM MK2206; Sigma-Aldrich, St Louis, MI, USA) to inhibit cell migration, or ROCK inhibitor (10 µM Y27632; Sigma-Aldrich) to accelerate cell migration post wounding. Multiple time lapse 1620 by 1176 px image sequences of wounded cells were captured with a magnification of 1.33 µm/px at 30-min intervals over 10.5 h (22 frames) using IncuCyte^®^ ZOOM (Essen Bioscience) [5]. We refer to image sequences containing Akt-inhibited, untreated and ROCK-inhibited cells as types of experiments: delayed, control and accelerated, respectively.

#### 2.1.2. CTMC Dataset

Unlike the long-existing Cell Tracking Challenge [55], the recently and publicly released CTMC dataset [36] is the only established cell tracking benchmark dataset that is designed for cell trackers utilizing bounding boxes for cell detection instead of performing cell segmentation. Hence, we were able to use the CTMC dataset as an independent validation dataset for EPIC. It consists of 86 diverse image sequences/videos for 14 different cell lines from animals, such as humans and rabbits, and features various cell types, such as myoblasts, fibroblasts and epithelial cells. The 320 by 400 px videos were collected over 300 to 4440 s (depending on the video) using the Nikon TE2000 Differential Interference Contrast imaging modality at 30 second intervals and at an approximate resolution of 0.35 µm/px and fully annotated with bounding boxes.

### 2.2. Algorithm

We trained a state-of-the-art AI system based on Vision Transformers to detect low-resolution airway epithelial cell nuclei and whole cells in our wound repair dataset and the CTMC dataset, respectively, using bounding boxes. We then developed a custom algorithm capable of tracking cells at low resolution and low framerate. The algorithm extracts two appearance and four motion features from cells detected in every frame of an image sequence. The extracted features are used to link cells with the same identity across frames with a custom multi-stage tracklet association and tracklet cleaving strategy based on combinatorial optimization [56,57,58]. We also developed a custom algorithm that can automatically identify the leading edges in wound repair images by analyzing the cell densities across the image plane. Full method details are given in the Supplementary Materials (Supplementary Methods, Additional File S1).

### 2.3. Performance Evaluation

We compared EPIC’s cell tracking performance in our wound repair dataset to manual cell tracking, as previously described [5]. We also compared EPIC’s cell tracking performance in our wound repair and the CTMC dataset to that of Viterbi [59] (offered as part of the Baxter Algorithms package [60]) and DeepSORT [61], the best performing publicly available automated trackers of their category benchmarked by Anjum and Gurari in the CTMC [36]. Full details of our comparisons and statistical inference are given in the Supplementary Materials (Supplementary Methods, Additional File S1).

## 3. Results and Discussion

### 3.1. Dataset Comparison

**Figure 1:**
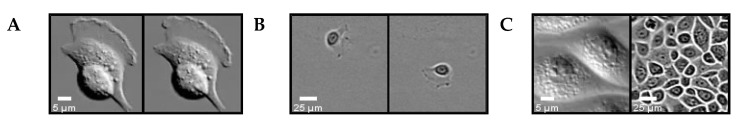
CTMC and wound repair dataset comparison. (**A**,**B**): A cell detected in the CTMC (**A**) and wound repair (**B**) dataset in two consecutive frames (left and right panels). Due to higher framerate, the cell displacement in panel (**A**) is almost unnoticeable compared to the cell in panel (**B**) captured at low framerate, which moved far away. (**C**): Equal sized image crops showing the difference in cell density between the CTMC (left) and wound repair dataset (right).

Our wound repair dataset was 380% lower resolution (1.33 µm/px) than the CTMC dataset (0.35 µm/px). Furthermore, all cells in our dataset were classified as small objects (<32 by 32 bounding box area) [44]. The CTMC dataset also contained cells under the small object category, as well the medium (>32 by 32 and <96 by 96 bounding box area) and large object (>96 by 96 bounding box area) categories [44]. Our dataset had over 65 times lower framerate (only 0.03 frames/min) than the CTMC dataset (2 frames/min) (Figure 1a,b). The average density in the CTMC dataset was approximately 13, at most ~28, cells per frame [36]. Conversely, each of our experiments had more than 70 times as many cells per frame, with over 1000 cells per frame (Figure 1c). These statistics reinforce the low-resolution, low-framerate and high-density nature of our wound repair dataset as is characteristic of data generated in high-throughput drug screening wound repair experiments.

### 3.2. Wound Repair Dataset

#### 3.2.1. Cell Detection

**Figure 2:**
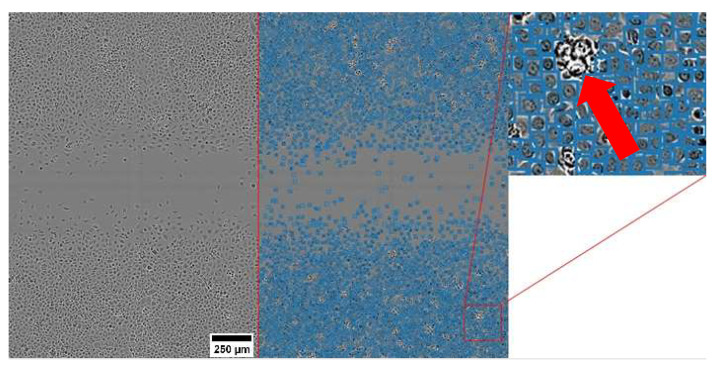
EPIC cell detection in a low-resolution and high-density wound repair image of the control experiment type. Left panel: unlabeled raw image, middle panel: same image with cell detections marked with blue bounding boxes, and right panel: enlarged region of middle panel showing accurate cell detections (blue boxes) and a large unlabeled region of cell debris (indicated by red arrow).

The training of the cell detection model was completed in ~30 min. The model achieved average precision and recall values of 87% and 91%, respectively. These metrics can be interpreted as the model correctly detecting ~90% of cells in each image. Figure 2 shows the detection performance on a full-sized wound repair image where most cells were detected as evidenced by the many bounding boxes (Figure 2, middle panel). These bounding boxes were well localized (Figure 2), which is notable given that the low-resolution and high-density cells are classified as small objects and are hence challenging to detect [44]. Importantly, the model was able to ignore cell debris (Figure 2, red arrow in inset), reinforcing EPIC’s ability to robustly learn the appearances of cells.

#### 3.2.2. Cell Tracking

As shown in Table 1, EPIC tracked over a hundred detected cells from the 1st to the 22nd frame without fragmentation in each of the nine total tested delayed, control and accelerated experiments (with three technical replicates per experiment type). From those cell tracks, we randomly sampled 20 leading edge cell tracks per experiment for wound repair analysis as is standard in the literature [5].

In contrast, DeepSORT failed to track any cells from the 1st to the 22nd frame without fragmentation in the same experiments, and with no cells tracked in the initial frames, no leading edge cell tracks could be defined and hence sampled for wound repair analysis (Table 1).

Viterbi had the most variable cell tracking performance in the same experiments, tracking anywhere from 0 to 2726 cells from the 1st to the 22nd frame without fragmentation depending on the experiment (Table 1). We noted that experiments with many (≥1883) or few (≤37) cell tracks (Table 1, Column 6) corresponded to experiments with lower (≤2283) or higher (≥3238) average numbers of cells per frame (as detected by EPIC), respectively. We posit that Viterbi’s non-AI-based image segmentation algorithm struggled to resolve the low-resolution high-density cells, a previously mentioned limitation of the approach, resulting in reduced numbers of generated cell tracks compared to experiments with lower cell density. Nevertheless, we were able to sample the 20 leading edge cell tracks for wound repair analysis in five of the experiments. Viterbi only generated 37 and 13 cell tracks in the control B and delayed C experiments among which there were only four and two leading edge cell tracks, respectively, that could be sampled for wound repair analysis. Viterbi failed to generate any cell tracks in the remaining control A and C experiments (Table 1).

*Wound Repair Analysis*

**Figure 3:**
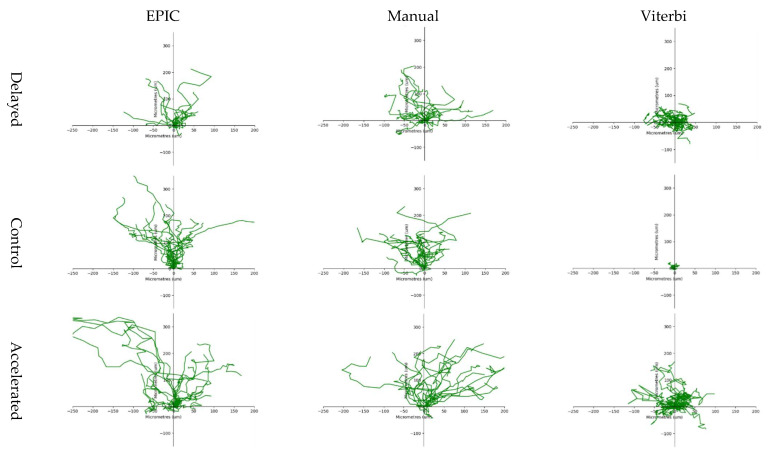
Cell trajectories of the leading edge cells tracked using manual cell tracking, EPIC and Viterbi in a delayed, control and accelerated experiment. Cell trajectories generated using manual cell tracking and EPIC indicate that cells primarily migrated in a positive vertical direction towards the wound region. In contrast, cell trajectories generated using Viterbi do not resemble leading edge cell tracks, instead suggesting that cells moved in more dispersed horizontal and vertical directions, including away from the wound area, contradicting EPIC and manual cell trajectories.

The sampled leading edge cell tracks that were generated by EPIC were largely similar to 20 randomly selected and manually tracked leading edge cells in the corresponding experiments, while sampled leading edge cell tracks generated by Viterbi were largely dissimilar to those manual cell tracks (Figure 3). Unlike Viterbi, both manual cell tracking and EPIC generated visibly shorter tracks for delayed experiments (Figure 3, top row) and longer tracks for accelerated experiments (Figure 3, bottom row) as compared to control (Figure 3, middle row), indicating that overall Viterbi cell tracks did not resemble the migration patterns expected of leading edge cells.

**Figure 4:**
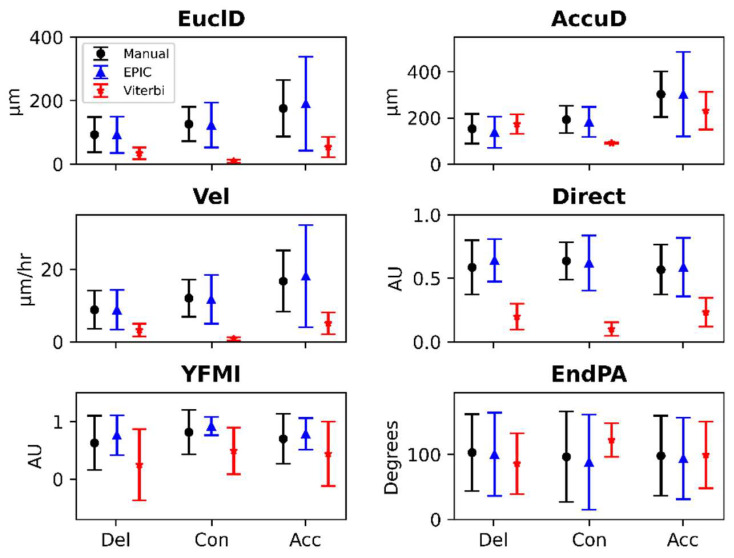
Cell migration metrics produced by manual cell tracking, EPIC and Viterbi. Various shapes and bars represent the mean and standard deviations, respectively, of cell migration metrics produced by manual (black circle), EPIC (blue triangle) and Viterbi (red star) cell tracking in delayed (Del), control (Con) or accelerated (Acc) experiments.

We used the manually and automatically generated leading edge cell tracks sampled from the nine total tested delayed, control and accelerated experiments to compute six cell migration metrics: Euclidean distance, accumulated distance, velocity, directionality, Y-forward migration index and end point angle. Cell migration metrics produced by manual cell tracking and EPIC were similar and both were different from metrics produced by Viterbi (Figure 4). The cell migration metrics of EPIC, Viterbi and manual cell tracking are shown in Figure 4 and Supplementary Table S3 (Additional File S1).

**Figure 5:**
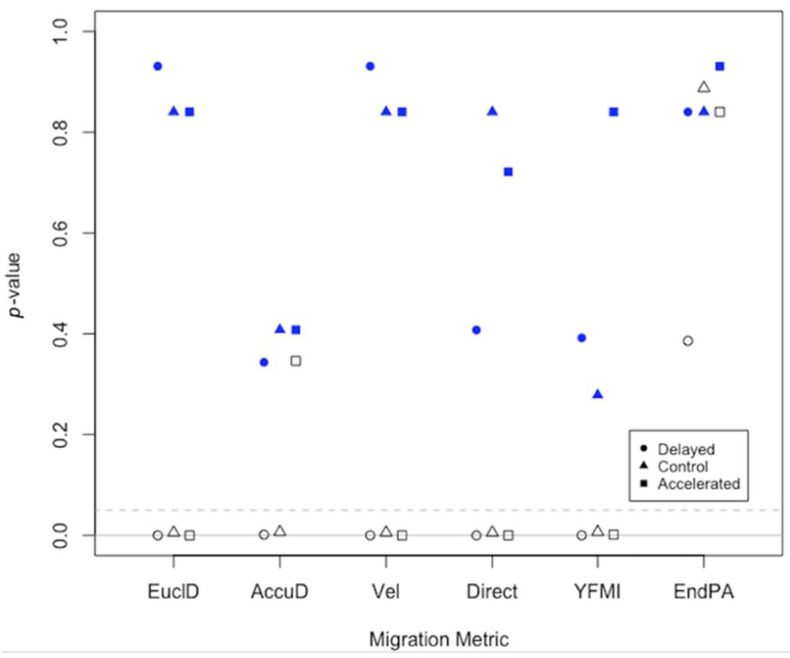
Comparison of the cell migration metrics produced by EPIC and Viterbi to manual cell tracking. Each symbol represents a *p*-value for pairwise comparisons of the sampled cell tracks from EPIC and manual cell tracking (blue filled) and Viterbi and manual cell tracking (black empty) for the delayed, control and accelerated experiments. We performed pairwise comparisons using two-sample Wilcoxon–Mann–Whitney tests. The statistical significance level was set to *p* < 0.05 (indicated by the dashed grey line). The solid grey line indicates y = 0. Metrics are shown in the following order and are abbreviated for clarity in the figure: Euclidean distance, accumulated distance, velocity, directionality, Y-forward migration index and end point angle.

Statistical comparison of the cell migration metrics produced by EPIC cell tracks indicated that there were no statistically significant differences to those produced by manual cell tracking for all metrics (Figure 5; Supplementary Tables S4 and S5, Additional File S1). On the other hand, we obtained mostly highly statistically significant differences when comparing Viterbi and manual cell tracks, indicating vast inaccuracies in metrics produced by Viterbi (Figure 5; Supplementary Tables S4 and S6, Additional File S1).

We found that wound repair analysis outcomes of untreated and drug-treated cells according to EPIC were equivalent to those of the current gold standard method, manual cell tracking, while generating at least 20 leading edge cell tracks across the 22 image frames without any user labor required. In contrast, Viterbi did not consistently track at least 20 leading edge cells across 22 frames. Additionally, Viterbi produced wound repair outcomes contradicting the expectations for untreated and drug-treated cells, and many of Viterbi cell tracks were visually incorrect. For instance, closer inspection revealed that, unlike EPIC (Supplementary Figure S5, Additional Files S1 and S2), many of Viterbi cell tracks did not correspond to real cells (Supplementary Figure S5, Additional Files S1 and S3). These results reinforce the challenging nature of the low-resolution, low-framerate and high cell density wound repair dataset even for well-established automated trackers.

#### 3.2.3. Automated Leading Edge Identification (EPIC)

**Figure 6:**
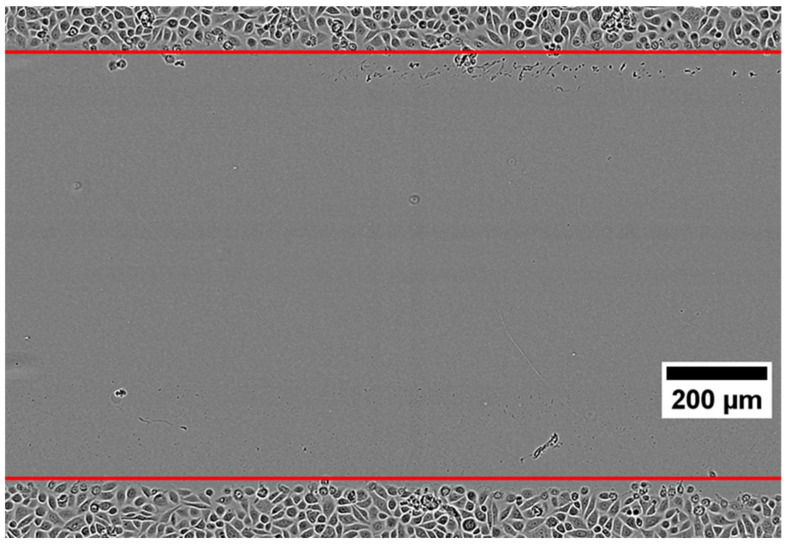
Automatically identified leading edges visualized as two horizontal red lines in the first frame of a control experiment.

EPIC automatically identified the leading edges of the wound in all experiments (Figure 6). By visual inspection, automatically detected leading edges were well localized even in the presence of significant cell debris. Additionally, we have shown that manual cell tracks sampled with respect to manually defined leading edges are comparable to cell tracks generated by EPIC and sampled with respect to automatically defined leading edges, reinforcing the accuracy of automated leading edge identification (Figure 6).

#### 3.2.4. Runtimes

EPIC detected and tracked thousands of cells in all the nine experiments in ~20 min (Table 2). In contrast, Viterbi and DeepSORT processed the same experiments in ~3 h and ~30 h, respectively (Table 2).

The tracking of hundreds of small objects in an image sequence is a challenging task outside the intended use cases of most existing trackers. Accordingly, EPIC’s efficient design and performance enhancements, such as advanced multicore processing, resulted in magnitudes faster processing times than Viterbi and DeepSORT. We anticipate Viterbi and DeepSORT’s long processing times to render these methods infeasible for use in high-throughput drug screening pipelines involving thousands of experiments. Although manual cell tracking is infeasible for high-throughput drug screening, the 20 cells from each delayed or control experiment were manually tracked within 10 min. Cells from each accelerated experiment were manually tracked within 30 min due to increased difficulty tracking fast-moving cells at the low framerate. The total manual tracking time for all experiments (180 cells) was 2.5 h.

### 3.3. CTMC Dataset

EPIC outperformed Viterbi and DeepSORT in cell tracking accuracy on the CTMC dataset (Supplementary Materials, Additional Files S1 and S4). Therefore, in addition to accurately tracking hundreds of detected cells in our wound repair dataset, EPIC also accurately detected and tracked cells in the CTMC dataset, which featured a higher framerate, higher resolution, 14 different cell lines and cells with extended cytoplasm. The sustained cell tracking accuracy of EPIC and its outperformance of other automated tools in the distinctly different and challenging dataset indicates robust underlying detection and tracking methods, and a system that is not limited to airway epithelial repair for children with asthma but can be applied in other cellular contexts.

### 3.4. Software Features and Report

We utilized object orientated software design principles, such as the Factory Method creational software design pattern, to allow developers to easily substitute custom object detectors and even tracking algorithms into EPIC to better suit other use cases, allowing for a highly flexible system. For researchers using EPIC ‘out-of-the-box’, it is a cross platform application that is simple to use through a command line or graphical user interface. Through the available commands, users can perform object detection, tracking and analysis of time lapse images in common formats, such as TIFF and JPEG. EPIC also automatically performs tracking and migration analyses of all cells in wound repair experiments. Generated cell tracks can be exported in multiple formats, such as ImageJ Manual Tracking File [62] and MOTChallenge [63] formats, for further external analyses. Importantly, EPIC generates a HTML report containing cell migration metrics, publication-ready figures such as cell trajectory plots, images and videos visualizing cell detections and tracks, and statistics such as the number of detected cells per frame (Additional File S5). Overall, EPIC can easily integrate into drug screening pipelines ‘out-of-the-box’ and is straightforward to use for non-programmers.

## 4. Conclusions

EPIC automatically tracked unstained cells (including drug-treated and untreated control) as accurately as manual cell tracking in a challenging low-resolution, low-framerate and high cell density dataset at higher volume and speed. This is unlike tested publicly available automated trackers, which underperformed on such a challenging dataset and hence cannot be reliably used for high-throughput wound repair analyses. EPIC also outperformed the same trackers on the diverse and challenging CTMC dataset, which includes 14 different cell lines, reinforcing that EPIC is not limited to airway epithelial repair for children with asthma but can be applied in other cellular contexts. EPIC tracks cells detected using state-of-the-art AI-based Vision Transformers and bounding boxes with a custom tracking algorithm. This results in highly accurate cell tracking with decreased labor and time necessary for training data generation compared to widely used segmentation-based approaches. We expect our open-source and easy-to-use software to enable high-throughput drug screening targeting airway epithelial repair for children with asthma.

## Figures and Tables

**Table 1:** A summary of the number of cells tracked from the 1st to the 22nd frame without fragmentation by EPIC, DeepSORT and Viterbi.

	EPIC		DeepSORT		Viterbi	
Experiment (Replicate)	Total Cell Tracks	Sampled Cell Tracks	Total Cell Tracks	Sampled Cell Tracks	Total Cell Tracks	Sampled Cell Tracks
Accelerated (A)	127	20	0	0	2046	20
Accelerated (B)	236	20	0	0	2008	20
Accelerated (C)	211	20	0	0	2019	20
Control (A)	783	20	0	0	0	0
Control (B)	539	20	0	0	37	4
Control (C)	586	20	0	0	0	0
Delayed (A)	146	20	0	0	1883	20
Delayed (B)	725	20	0	0	2726	20
Delayed (C)	1006	20	0	0	13	2

**Table 2:** Total runtimes for EPIC, DeepSORT and Viterbi in the 9 experiments.

	EPIC	DeepSORT	Viterbi
Total Running Time	20 min	30 h	3 h

## Data Availability

The datasets generated and/or analyzed during the current study are available in the developed software’s repository along with the code, https://github.com/AlphonsG/EPIC-BBox-Cell-Tracking (accessed on 9 February 2022), and MOTChallenge website, https://motchallenge.net/data/CTMC-v1/ (accessed on 9 February 2022).

## Supplementary Materials

The following supporting information can be downloaded at: https://www.mdpi.com/article/10.3390/jpm12050809/s1, Additional Files S1–S5.
