# Supplementary material for: AI-Driven Cell Tracking to Enable High-Throughput Drug Screening Targeting Airway Epithelial Repair for Children with Asthma
Source: J Pers Med. 2022 May 17;12(5):809. doi: 10.3390/jpm12050809 (PMC9146422; doi:10.3390/jpm12050809)
Supplement: Supplementary file 1 [file jpm-12-00809-s001.zip › Supplementary Materials/Additional File 1.pdf]

Supplementary Materials

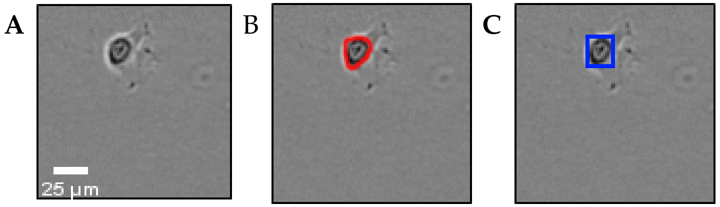

Supplementary Figure S1. Comparison of cell segmentation and cell detection using bounding boxes. A: A single airway epithelial cell. B: The same cell that has been segmented by carefully drawing the exact outline of the cell nucleus using a mouse with the left mouse button held down. C: The same cell that has been enclosed with a bounding box by clicking twice only with a mouse to specify the upper-leftmost and lower-rightmost corners of the bounding box.

Supplementary Methods

1. Algorithm

We used four steps in the design of our tracking algorithm: detection, feature extraction, affinity, and association [1].

a. Detection

We utilized an artificial intelligence (AI)-based approach for cell detection using bounding boxes. Transformers [2] are widely used for natural language processing and have recently demonstrated high accuracy in computer vision tasks as an alternative to conventional convolutional neural networks (CNNs) [3]. We therefore selected the state-of-the-art Swin Transformer [3] with feature pyramid networks [4] as the backbone of our cell detection framework. The framework consists of Cascade R-CNN [5], a multi-

stage extension of Faster R-CNN, which features a region proposal network (RPN) and region-based CNN for multi-class object detection [6]. The backbone and framework were sourced and configured using MMDetection [7]; an open-source object detection toolbox based on PyTorch [8]. Furthermore, we perform cell detection in inference images using a 224 x 244 px sliding window with a 60 px stride and non-maximum suppression threshold of 0.1.

b. Feature Extraction and Affinity

We chose a unique combination of appearance and motion features to extract from cells detected in an image sequence for reliable performance. The extracted features are used to calculate the affinity, or association likelihood, between cells, that is: the probability that two cells detected in different frames are the same cell.

Appearance Features

We extract two appearance features from cells: *Grayscale Histograms* (h) and *Structural Similarity Index (SSIM)* (s). The degree of intersection between the grayscale histograms [9] extracted from two detected cells, which ranges from 0 to 1, is used as the grayscale histogram association likelihood, p_h. The SSIM is a measure of image similarity based on the structural qualities of two images using local patterns of pixel intensities [10]. The SSIM between two detected cells, which ranges from 0 to 1, is used as the SSIM association likelihood, p_s, to capture the spatial relationships between pixels which are disregarded by grayscale histograms. Grayscale histograms and the SSIM are reliably performing appearance features as even with low-framerate wound repair image sequences, the appearance of detected cell nuclei should be relatively consistent across frames despite potential cytoplasm deformations.

Motion Features

We extract four motion features from cells: *Intersection over Union (IoU)* (u), *Euclidean Distance* (e), *Motion Vectors* (m) *and Temporal distance* (t). The IoU, also known as the Jaccard index, of two detected cells of the same identity in different frames is expected to be discriminatively large compared to unidentical cells. The IoU between cells is used as the IoU association likelihood, p_u. In addition to the IoU, which can fail at low framerates or with fast moving objects, we use the euclidean distance between

the centres of two detected cells to model cell spatial proximity. The euclidean distance association likelihood, p_E, is calculated as one minus the euclidean distance between cells, normalized by a maximum distance specified in Supplementary Table S2 and S3.

We also incorporate a constant "heading" assumption into a motion feature, that uses the motion vectors between the previous positions of a cell in earlier frames, to avoid linking cells that deviate drastically from the current trajectory (Supplementary Figure S2). The motion vector association likelihood, p_m, is calculated as the angle θ divided by 180 (Supplementary Figure S2).

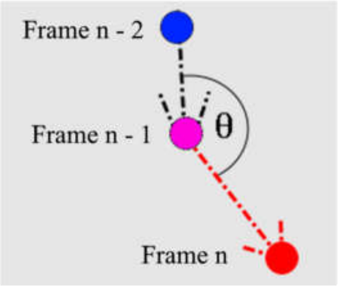

Supplementary Figure S2. The motion vectors (red and black arrows) between potentially identical cells in 3 different frames (blue, pink, and red circles). The angle of travel θ is computed using cells across 3 frames and is always less than or equal to 180 degrees.

We use the temporal distance, defined as one plus the number of frames between two detected cells, to avoid linking cells separated by many frames. The temporal distance association likelihood, p_t, is calculated as one minus the temporal distance between cells, normalized by a maximum distance specified in Supplementary Table S2 and S3.

c. Association

We use a *tracklet*-based optimization approach as the basis of our association algorithm [11–13]. We first define the joint association likelihood, P, as:

$$P = \begin{cases} \frac{1}{N}\sum_{i \in I} w_i p_i & if\ \forall i \in I, \quad p_i \geq t_i \\ \text{UNDEFINED} & otherwise \end{cases} \qquad Equation\ 1$$

where $I = \{h,\ s,\ u,\ e,\ m,\ t\}$, w_i $(0\ \leq\ w_i\ \leq\ 1)$ is the weight of each feature association likelihood, $\mathrm{t_i}$ is the minimum threshold value $(0\ <\ \mathrm{t_i}\ \leq\ 1)$ that the corresponding p_i has to meet, $N = |\forall i \in I,\ w_i > 0|$ and $0 \leq P \leq 1$. The joint association likelihood averages the weighted association likelihoods of each feature to produce one combined measure of cell similarity. Minimum threshold values cause the joint association likelihood, P, to be undefined if a particular appearance or motion feature produces a very poor association likelihood.

In our algorithm, we first detect cells in every frame of an image sequence of length M and then iterate frame 1 up to frame $M - 1$. At each iteration, the current frame f_m and all proceeding frames $F = \{f_{m+1}, \dots, f_{m+a}\}$ within α (frame look-ahead parameter) $(1 \leq\ a\ \leq \mathrm{M} - 1)$ frame(s) of f_m are processed. Joint association likelihoods, P, between each cell from f_m and each cell from each frame in F, within β (neighbourhood distance parameter) euclidean distance of each other, are then computed. We identify the optimal assignment of cells in the current frame to cells in the proceeding frame(s) by constructing a cost matrix with inverted joint association likelihoods, $\frac{1}{P}$, between the cells and applying the Hungarian algorithm [14]. An example of the cost matrix for 2 temporally adjacent frames, each containing 3 detected cells, with the frame look ahead parameter (a) set to 1 is shown in Supplementary Table S1. The minimum cost assignment solution resolves cells x_1 and x_4, cells x_2 and x_5, and cells x_3 and x_6 as most likely having the same identity (bolded).

Supplementary Table S1. Hypothetical example of a cost matrix containing the inverted joint association likelihoods, $\frac{1}{P}$, between 3 cells in two adjacent frames.

	Cell x_4	Cell x_5	Cell x_6
Cell x_1 f_m f_{m+1}	**0.3**	0.9	0.9
Cell x_2	0.9	**0.4**	0.8
Cell x_3	*0.1*	0.9	**0.2**

$\boldsymbol{f_m}$**:** current frame**,** $\boldsymbol{f_{m+1}}$**:** next frame**.** The bolded costs represent the original cost matrix solution. The italicized cost represents a suboptimal association in this solution. The underlined costs represent a more ideal final cost matrix solution for object tracking after applying our cost matrix modification strategy.

By performing a one-to-one association between every row and column in the cost matrix, cells in the current frame are linked to cells in the following frame(s), resulting in partial cell tracks, or tracklets. A row and column are not assignable if the corresponding joint association likelihood P is undefined, and one row or column in the largest dimension is guaranteed to be unassigned in the case of a non-square cost matrix.

A limitation of tracking methods based on the Hungarian algorithm is that a solved cost matrix can contain row assignments with columns that do not have the lowest cost since every row and column must be assigned where possible. For example, in Supplementary Table S1, cell x_3 has a poorer assignment (higher cost) with cell x_6 (column 3/bolded) than with cell x_4 (column 1/italicized). Since poor assignments may result in incorrect cell tracks, we use a unique strategy to modify the solved cost matrix by first removing all poor assignments from the matrix. We then iterate each row (representing a cell from the current frame) that contained a poor assignment in the previously solved matrix for correction. At each iteration, if the column (representing a cell from a future frame) with the lowest cost is not assigned to any row, we reassign it to the current row. If the column with the lowest cost is already assigned to a different row, but that existing cost is greater than that with the current row, we remove the column's existing assignment and re-assign the column to the current row. For example, in Supplementary Table S1, the cost of cell x_4's existing assignment with cell x_1 (0.3/bolded) is greater than cell x_4's potential assignment with cell x_3 (0.1/italicized), hence we remove the former cell assignment and replace it with the latter. With this approach, the final cost matrix solution in Supplementary Table S1 involves the underlined assignments (cell assignments with the lowest cost only), which are generated tracklets, while the first row (cell x_1) is unassigned to any column (cell), which are cleaved tracklets.

We continue iterating through all frames in the image sequence while first associating cells and then performing tracklet cleaving at each frame to finally produce both extended and cleaved cell tracklets. To link some of the previously cleaved tracklets, or unlinked cell tracks due to missed cell detections, and benefit from multi-frame association [15], we allow the entire image sequence to be traversed multiple times, while re-performing association and cleaving of previously generated tracklets using different $w_i, t_i, \alpha, and\ \beta$ parameters. All chosen tracking parameter values are provided in Supplementary Table S1 and S2. We refer to the initial image sequence traversal as the primary run, and the rest as secondary runs. Our algorithm terminates when the latest run does not result in any tracklets being linked or cleaved.

d. Automated Leading Edge Identification

EPIC automatically identifies the leading edges using two sliding windows that each initialize 24 strides from the top and bottom of the first frame of a wound repair image sequence and slide towards the center of the image. We set the stride equal to the average diameter of all detected cells in the image. The sliding window width is set to the image width and window height to one stride. The algorithm for leading edge identification is as follows: let x be an ordered list of the numbers of cells, or cell densities, within a window for the current and all previous window positions. If the mean(x) – 3 * std(x) < 0, where mean(x) and std(x) is the average and standard deviation of the values in x, respectively, and the cell density at the current window position is greater than that of the previous position, we move the window to the next position by one stride. If the previous condition fails and the cell density at the current window position is less than the mean(x) – 2 * std(x), we define the position of a leading edge as the bottom/top (for the upper/lower leading edge, respectively) of the current window.

2. Training

We trained our cell detection model using initial model weights from the ImageNet-1K/COCO 2017 [16, 17] pre-trained Swin Transformer (Swin-B variant)/Cascade R-CNN backbone/framework combination [7].

Wound Repair Dataset

To train the cell detection model, we used training and testing sets of 21 (70%) and 9 (30%) images, respectively. Furthermore, we split our training set into 15 (70%) and 6 (30%) training and validation images, respectively. We selected training images from a control experiment and selected validation images from another independent control experiment. We formed the 9-image testing set by selecting three sets of 3 images from an independent delayed, control and accelerated experiment, with one set per experiment type. All images were randomly selected from all frames of the respective experiment, without replacement, and once selected were cropped at a random position into a single 224x224 px sub image. Cropped images contained on average 80 cells and all nuclei were annotated with bounding boxes. Our training settings were modified

from those of the default MMDetection Swin Transformer (Swin-B) configuration: no image resizing; AdamW optimizer [18] with initial learning rate of 0.0001, weight decay of 0.05, and batch size of 1; 100 epochs. We adjusted the RPN base anchor sizes and ratios to compensate for low-resolution cells and increased the number of RPN and R-CNN proposals to compensate for high cell density. We also included the following online image augmentations: random brightness, contrast, blur, gaussian noise, 90°, 180° and 270° image rotations, and vertical and horizonal image flips using the Albumentations library [19]. We assessed cell detection performance in the testing set using average precision (AP) and recall (AR) at an IoU threshold of 0.5.

Cell Tracking with Mitosis Detection Challenge (CTMC) Dataset

We trained our cell detection model using the official CTMC training dataset, which was released with ground truth bounding box annotations. We randomly selected 70% of the videos from each of the 14 cell lines to form the training set and combined the remaining videos (30%) as the validation set. We discarded all frames corresponding to frame numbers that were not multiples of 15 from each video. We used the official CTMC testing dataset as the testing set, which has unreleased ground truth annotations. We used the same training settings as for the wound repair dataset with the following changes: batch size of 8; 36 epochs; and no 90° and 270° image rotations due to non-square images.

3. Performance Evaluation

We performed training and evaluation on an Ubuntu 21.04 system with an AMD Ryzen™ 7 5800X, NVIDIA GeForce RTX™ 3090, and 64 GB DDR4 RAM.

Wound Repair Dataset

For performance comparisons, we tracked cells in our wound repair dataset using DeepSORT [20] and Viterbi [21] (offered as part of the Baxter Algorithms package [22]), in addition to EPIC. Viterbi is the highest scoring publicly available segmentation-based cell tracker and DeepSORT is the highest scoring (with ground truth detections)

publicly available mainstream object tracker that uses bounding boxes benchmarked by Anjum and Gurari in the CTMC [23]. DeepSORT is a popular AI-based mainstream object tracker while Viterbi is a non-AI-based top performer in the Cell tracking Challenge (CTC) [24]. The settings used for all trackers are given in Tracker Settings.

Due to the large number of cells in a wound repair image sequence, it is physically impossible to generate the ground truth data required to quantify cell tracking performance using standard metrics such as Multiple Object Tracking Accuracy (MOTA) [25]. Additionally, these metrics are not necessarily relevant in determining a tool's applicability for automatically assessing the wound repair outcomes of drug treated cells relative to the current gold standard method, manual cell tracking.

For evaluation, we therefore followed the previously established protocol for wound repair analysis [26]: 20 leading edge cells, 10 on each wound edge, were first randomly selected and manually tracked from the 1st to the 22nd frame (10.5 hours) in 9 total delayed, control and accelerated experiments (with three technical replicates per experiment type). Furthermore, we automatically tracked all detected cells, again from the 1st to the 22nd frame, in the same experiments using EPIC, DeepSORT and Viterbi. As required by the wound repair analysis protocol, in each experiment we then randomly sampled 20 leading edge cells, 10 within 55 px behind each leading edge automatically identified by EPIC in the first frame of the corresponding experiment, that were tracked from the 1st to the 22nd frame without fragmentation using all three automated methods. We computed six standard cell migration metrics: *euclidean distance*, *accumulated distance*, *velocity*, *directionality*, *end point angle*, and *Y-forward migration index* (see Metric Definitions) from all automatically and manually generated leading edge cell tracks for statistical analyses.

To evaluate EPIC, DeepSORT, and Viterbi's wound repair analysis performance, we compared the cell migration metrics produced by each automated method to those produced by manual cell tracking in each experiment. Specifically, we performed pair-wise comparisons using non-parametric two-tailed Wilcoxon-Mann-Whitney tests. Non-parametric testing was used as two metrics (*end point angle* and *Y-forward migration index*) had non-Normal distributions, while two others (raw *euclidean distance and velocity*) were right skewed right and became left skewed after a log2 transformation. All p-values were adjusted using Benjamini-Hochberg multiple testing adjustment [27].

CTMC Dataset

We used EPIC to detect (with the sliding window disabled due to smaller images) and track cells, using the settings given in Tracker Settings, in the CTMC testing dataset and uploaded the generated cell track data to the CTMC evaluation server. We compared EPIC's reported cell tracking accuracy to that of Viterbi and DeepSORT, which

were previously benchmarked by Anjum and Gurari in the CTMC [23]. Tracking performance was compared using MOTA [25], which is perhaps the most widely used metric to evaluate a tracker's performance [25], and Tracking Accuracy (TRA) [28], which is the standard tracking metric in the CTC (see Metric Definitions).

4. Tracker Settings

DeepSORT settings for the wound repair dataset

DeepSORT [20] is a AI-based tracker that tracks objects detected using bounding boxes. To compare the accuracy of cell tracking only, we provided DeepSORT with cell detections generated by EPIC for all experiments. The tracking settings for DeepSORT were as follows: minimum detection confidence of 0.75 (as with EPIC); no minimum detection height; maximum appearance cosine distance of 0.2, no maximum size of the appearance descriptors gallery; and non-maximum suppression threshold of 1.

Viterbi settings for the wound repair dataset

Viterbi's [21] (v1.6.1) segmentation and tracking settings are shown in Supplementary Figures S3 and S4, respectively. Multicore processing was enabled for runtime benchmarking.

SegAlgorithm	Segment_localvariance
SegChannel	Ch1
SegBgSubAlgorithm	BgSub_median
SegMinArea	40
SegWatershed	intermediate
SegWSmooth	1
SegWHMax	0.01
LVSegRegionSize	2
LVSegThreshold	5
LVSegErodeSize	3

Supplementary Figure S3. Viterbi’s segmentation settings.

TrackXSpeedStd	12
pCnt0	0.2
pCnt1	0.7
pCnt2	0.1
pSplit	0.01
pDeath	0.01
TrackPAppear	0
TrackPDisappear	0

Supplementary Figure S4. Viterbi’s tracking settings.

EPIC settings for the wound repair and CTMC dataset

EPIC's tracking settings for the wound repair and CTMC datasets are shown in Supplementary Tables S1 and S2, respectively. We only tracked cells detected with confidence scores above 0.75 and 0.25 in the wound repair and CTMC dataset, respectively.

Supplementary Table S2. Wound repair dataset tracking settings.

**Run**	$\boldsymbol{\alpha}$	$\boldsymbol{\beta}$		**Feature ($\boldsymbol{i}$)**					
				$\boldsymbol{h}$	$\boldsymbol{s}$	$\boldsymbol{u}$	$\boldsymbol{e}$	$\boldsymbol{m}$	$\boldsymbol{t}$
**Primary**	1	45	$\boldsymbol{w_i}$	1	0.5	0.5	1	1	0
			$\boldsymbol{t_i}$	N.S	N.S	N.S	45 (13.5)*	N.S	N.S
**Secondary**	2	50	$\boldsymbol{w_i}$	1	0.5	0.5	1	1	1
			$\boldsymbol{t_i}$	N.S	N.S.	N.S.	50 (15)*	N.S.	2

$\boldsymbol{w_i}$: weight, $\boldsymbol{t_i}$: threshold, $\boldsymbol{h}$: grayscale histogram, $\boldsymbol{s}$: SSIM, $\boldsymbol{u}$: intersection over union, $\boldsymbol{e}$: euclidean distance, $\boldsymbol{m}$: motion vectors, $\boldsymbol{t}$: temporal distance, $\boldsymbol{\alpha}$: frame look-ahead parameter, $\boldsymbol{\beta}$: neighbourhood distance parameter, **N.S.**: not set. *Parameter values in brackets are used for cells in any frame that are behind one of the leading edge positions automatically detected by EPIC in the first frame of the respective image sequence. Cells within 15 px. of the image boundary in secondary runs are not associated to limit interference from cells entering and leaving the image plane.

Supplementary Table S3. CTMC dataset tracking settings.

Run	α	β		Feature (i)					
				h	s	u	e	m	t
**Primary**	1	10	w_i	1	0.5	1	1	1	0
			t_i	N.S	N.S	0.5	10	N.S	N.S
**Secondary**	5	20	w_i	1	0.5	1	1	1	1
			t_i	N.S	N.S.	0.5	20	N.S.	5

$\boldsymbol{w_i}$: weight, $\boldsymbol{t_i}$: threshold, $\boldsymbol{h}$: grayscale histogram, $\boldsymbol{s}$: SSIM, $\boldsymbol{u}$: intersection over union, $\boldsymbol{e}$: euclidean distance, $\boldsymbol{m}$: motion vectors, $\boldsymbol{t}$: temporal distance, $\boldsymbol{\alpha}$: frame look-ahead parameter, $\boldsymbol{\beta}$: neighbourhood distance parameter, **N.S.**: not set.

5. Metric Definitions

Detection Metrics

AP is the proportion of true cell detections to the total number of detected cells within an image, expressed as a percentage, averaged over all images. AR is the proportion of true cell detections to the total number of actual cells within an image, expressed as a percentage, averaged over all images. AP and AR scores can range between 0% and 100% and increase with better detection performance.

Tracking Metrics

TRA is how accurately each cell has been identified and tracked in successive frames and is based on the comparison of graphs representing the predicted and the ground truth cell tracks [23, 28]. TRA scores can range between 0 and 1 and increase with better tracking performance [23]. MOTA quantifies tracking accuracy by performing one-

to-one matching between the predicted detections and ground truth detections of tracks across frames and penalizing errors such as missing predictions [29]. MOTA scores can range between negative values and 100 and increase with better tracking performance [23].

Cell Migration Metrics

Euclidean distance measures the distance between start and end point of a cell trajectory.

$$\text{Euclidean Distance } (\mu\text{m}) = \sqrt{(x_{\text{final}} - x_0)^2 + (y_{\text{final}} - y_0)^2}$$

Where x_0, y_0 are the initial x and y coordinates of a cell and $x_{\text{final}}, y_{\text{final}}$ are the final x and y coordinates of a cell.

Accumulated distance measures the total length of a cell trajectory path.

$$\text{Accumulated Distance } (\mu\text{m}) = \sum_{i=1}^{n} \sqrt{(x_i - x_{i-1})^2 + (y_i - y_{i-1})^2}$$

Where, x_i, y_i are the x and y coordinates of a cell at the ith frame in a wound repair image sequence.

Velocity measures the rate at which a cell changes position.

$$\text{Velocity } (\mu\text{m/hr}) = \frac{\text{Euclidean Distance}}{\Delta t}$$

Where, Δt is the frame difference between the first and current frame in the cell track and the Euclidean distance is the Euclidean distance at the current position.

Directionality measures the straightness of an individual cell trajectory.

$$\text{Directionality (AU)} = \frac{\text{Euclidean Distance}}{\text{Accumulated Distance}}$$

Y-Forward Motion Index measures the migration of a cell towards/away from the wound.

$$\mathrm{Y-Forward\ Motion\ Index\ (AU)} = \frac{y_{\mathrm{final}}}{\mathrm{Euclidean\ Distance}}$$

Where, y_{final} is the final y coordinate of a cell.

Endpoint angle measures the angle of the final track coordinates with respect to the initial track coordinates.

$$\mathrm{Endpoint\ Angle\ } (\theta^{\circ}) = \cos^{-1}\left(\frac{y_{\mathrm{final}} - y_{0}}{\mathrm{Euclidean\ Distance}}\right)$$

Wound Repair Dataset Results

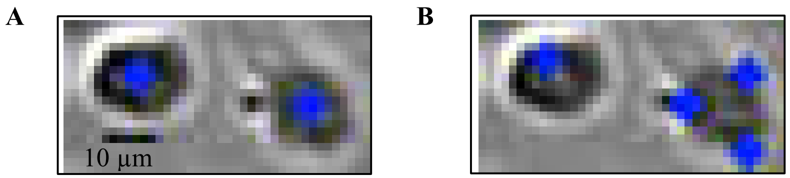

Supplementary Figure S5. Side by side view of the same image, cropped from the first frame in a delayed experiment, containing 2 cells for EPIC (A) and Viterbi (B) cell track comparison. A: EPIC correctly associates each cell track (blue dot) to each of the two cells. B: Viterbi correctly associates the left cell to a single cell track, but incorrectly associates the right cell with 3 cell tracks. Each blue dot represents the beginning of a cell track for each method.

Supplementary Table S4. Cell migration metrics produced by EPIC, Viterbi, and manual cell tracking.

Cell Migration Metric	Cell Tracking Method	Experiment Type					
		Delayed		Control		Accelerated	
		Mean	SD	Mean	SD	Mean	SD
Euclidean Distance (μm)	Manual	92.71	55.55	126.18	53.98	175.43	88.77
	EPIC	92.37	57.46	122.56	70.55	189.98	147.82
	Viterbi	33.72	18.27	8.89	4.52	53.28	31.79
Accumulated Distance (μm)	Manual	152.95	64.55	192.67	59.12	302.82	99.05
	EPIC	137.69	68.28	182.20	64.96	302.69	183.12
	Viterbi	172.61	41.87	90.65	4.15	231.12	81.38
Velocity (μm/hr)	Manual	8.83	5.29	12.02	5.14	16.71	8.45
	EPIC	8.80	5.47	11.67	6.72	18.09	14.08
	Viterbi	3.21	1.74	0.85	0.43	5.07	3.03
Directionality (AU)	Manual	0.59	0.21	0.64	0.15	0.57	0.20
	EPIC	0.64	0.17	0.62	0.22	0.59	0.23
	Viterbi	0.20	0.10	0.10	0.05	0.23	0.11
Y Forward Motion Index (AU)	Manual	0.63	0.47	0.81	0.38	0.70	0.43
	EPIC	0.76	0.34	0.92	0.16	0.78	0.27
	Viterbi	0.25	0.62	0.49	0.40	0.44	0.56
Endpoint Angle (Degrees)	Manual	102.71	59.10	96.25	69.29	97.77	61.60

	EPIC	100.02	63.97	88.11	73.26	93.70	62.68
	Viterbi	85.54	46.62	121.89	25.82	98.96	51.19

SD: standard deviation; AU: arbitrary units.

Supplementary Table S5. Benjamini-Hochberg adjusted p-values from the pairwise comparison of cell migration metrics produced by EPIC against manual cell tracking using two-sample Wilcoxon-Mann-Whitney tests, also shown in Figure 6.

Experiment Type	Euclidean Distance (µm)	Accumulated Distance (µm)	Velocity (µm/hr)	Directionality (AU)	Y-Forward Motion Index (AU)	Endpoint Angle (Degrees)
Delayed	0.93	0.34	0.93	0.41	0.39	0.84
Control	0.84	0.41	0.84	0.84	0.28	0.84
Accelerated	0.84	0.41	0.84	0.72	0.84	0.93

*Statistical significance level was set to $p<0.05$.

Supplementary Table S6. Benjamini-Hochberg adjusted p-values from the pairwise comparison of cell migration metrics produced by Viterbi against manual cell tracking using two-sample Wilcoxon-Mann-Whitney tests, also shown in Figure 6.

Experiment Type	Euclidean Distance (µm)	Accumulated Distance (µm)	Velocity (µm/hr)	Directionality (AU)	Y-Forward Motion Index (AU)	Endpoint Angle (Degrees)
Delayed	*0.00	*0.0013	*0.00	*0.00	*0.00	0.39
Control	*0.0054	*0.0066	*0.0054	*0.0054	*0.0067	0.89
Accelerated	*0.00	0.35	*0.00	*0.00	*0.0016	0.84

*Statistical significance level was set to $p<0.05$.

CTMC Dataset Results

EPIC achieved an average TRA of 0.50 (see official results, Additional File 4), outperforming Viterbi which achieved an average TRA of 0.39 [23]. EPIC achieved an average MOTA of 41.22 (see official results, Additional File 4), outperforming DeepSORT which achieved an average MOTA of -36.70 [23].

References

1. Ciaparrone G, Luque Sánchez F, Tabik S, Troiano L, Tagliaferri R, Herrera F. Deep learning in video multi-object tracking: A survey. Neurocomputing. 2020;381:61–88.

2. Vaswani A, Shazeer N, Parmar N, Uszkoreit J, Jones L, Gomez AN, et al. Attention is all you need. In: Proceedings of the 31st International Conference on Neural Information Processing Systems. Red Hook, NY, USA: Curran Associates Inc.; 2017. p. 6000–10.

3. Liu Z, Lin Y, Cao Y, Hu H, Wei Y, Zhang Z, et al. Swin Transformer: Hierarchical Vision Transformer using Shifted Windows. arXiv:210314030 [cs]. 2021.

4. Lin T, Dollár P, Girshick R, He K, Hariharan B, Belongie S. Feature Pyramid Networks for Object Detection. In: 2017 IEEE Conference on Computer Vision and Pattern Recognition (CVPR). 2017. p. 936–44.

5. Cai Z, Vasconcelos N. Cascade R-CNN: Delving Into High Quality Object Detection. In: 2018 IEEE/CVF Conference on Computer Vision and Pattern Recognition. 2018. p. 6154–62.

6. Ren S, He K, Girshick R, Sun J. Faster R-CNN: Towards Real-Time Object Detection with Region Proposal Networks. IEEE Transactions on Pattern Analysis and Machine Intelligence. 2015;39.

7. Chen K, Wang J, Pang J, Cao Y, Xiong Y, Li X, et al. MMDetection: Open MMLab Detection Toolbox and Benchmark. CoRR. 2019;abs/1906.07155.

8. Paszke A, Gross S, Massa F, Lerer A, Bradbury J, Chanan G, et al. PyTorch: An Imperative Style, High-Performance Deep Learning Library. In: Advances in Neural Information Processing Systems. Curran Associates, Inc.; 2019.

9. Swain MJ, Ballard DH. Color indexing. International Journal of Computer Vision. 1991;7:11–32.

10. Zhou Wang, A. C. Bovik, H. R. Sheikh, E. P. Simoncelli. Image quality assessment: from error visibility to structural similarity. IEEE Transactions on Image Processing. 2004;13:600–12.

11. Roth M, Bäuml M, Nevatia R, Stiefelhagen R. Robust multi-pose face tracking by multi-stage tracklet association. In: Proceedings of the 21st International Conference on Pattern Recognition (ICPR2012). 2012. p. 1012–6.

12. Wang G, Wang Y, Zhang H, Gu R, Hwang J-N. Exploit the Connectivity: Multi-Object Tracking with TrackletNet. In: Proceedings of the 27th ACM International Conference on Multimedia. New York, NY, USA: Association for Computing Machinery; 2019. p. 482–90.

13. Ma C, Yang C, Yang F, Zhuang Y, Zhang Z, Jia H, et al. Trajectory Factory: Tracklet Cleaving and Re-Connection by Deep Siamese Bi-GRU for Multiple Object Tracking. In: 2018 IEEE International Conference on Multimedia and Expo (ICME). 2018. p. 1–6.

14. Kuhn HW. The Hungarian Method for the Assignment Problem. In: Jünger M, Liebling TM, Naddef D, Nemhauser GL, Pulleyblank WR, Reinelt G, et al., editors. 50 Years of Integer Programming 1958-2008: From the Early Years to the State-of-the-Art. Berlin, Heidelberg: Springer Berlin Heidelberg; 2010. p. 29–47.

15. Smal I, Meijering E. Quantitative comparison of multiframe data association techniques for particle tracking in time-lapse fluorescence microscopy. Medical Image Analysis. 2015;24:163–89.

16. Lin T-Y, Maire M, Belongie S, Hays J, Perona P, Ramanan D, et al. Microsoft COCO: Common Objects in Context. In: Fleet D, Pajdla T, Schiele B, Tuytelaars T, editors. Computer Vision – ECCV 2014. Cham: Springer International Publishing; 2014. p. 740–55.

17. Deng J, Dong W, Socher R, Li L-J, Li K, Fei-Fei L. ImageNet: A large-scale hierarchical image database. In: 2009 IEEE Conference on Computer Vision and Pattern Recognition. 2009. p. 248–55.

18. Loshchilov I, Hutter F. Decoupled Weight Decay Regularization. In: ICLR. 2019.

19. Buslaev A, Iglovikov VI, Khvedchenya E, Parinov A, Druzhinin M, Kalinin AA. Albumentations: Fast and Flexible Image Augmentations. Information. 2020;11:125.

20. N. Wojke, A. Bewley, D. Paulus. Simple online and realtime tracking with a deep association metric. In: 2017 IEEE International Conference on Image Processing (ICIP). 2017. p. 3645–9.

21. Magnusson KEG, Jaldén J, Gilbert PM, Blau HM. Global Linking of Cell Tracks Using the Viterbi Algorithm. IEEE Transactions on Medical Imaging. 2015;34:911–29.

22. Magnusson K. klasma/BaxterAlgorithms. 2021. https://github.com/klasma/BaxterAlgorithms. Accessed 17 Sep 2021.

23. Anjum S, Gurari D. CTMC: Cell Tracking with Mitosis Detection Dataset Challenge. In: 2020 IEEE/CVF Conference on Computer Vision and Pattern Recognition Workshops (CVPRW). 2020. p. 4228–37.

24. Maška M, Ulman V, Svoboda D, Matula P, Matula P, Ederra C, et al. A benchmark for comparison of cell tracking algorithms. Bioinformatics. 2014;30:1609–17.

25. Milan A, Leal-Taixe L, Reid I, Roth S, Schindler K. MOT16: A Benchmark for Multi-Object Tracking. arXiv:160300831 [cs]. 2016.

26. Iosifidis T, Sutanto EN, Buckley AG, Coleman L, Gill EE, Lee AH, et al. Aberrant cell migration contributes to defective airway epithelial repair in childhood wheeze. JCI Insight. 2020;5.

27. Benjamini Y, Hochberg Y. Controlling the False Discovery Rate: A Practical and Powerful Approach to Multiple Testing. Journal of the Royal Statistical Society Series B (Methodological). 1995;57:289–300.

28. Ulman V, Maška M, Magnusson KEG, Ronneberger O, Haubold C, Harder N, et al. An objective comparison of cell-tracking algorithms. Nat Methods. 2017;14:1141–52.

29. Luiten J, Ošep A, Dendorfer P, Torr P, Geiger A, Leal-Taixé L, et al. HOTA: A Higher Order Metric for Evaluating Multi-object Tracking. International Journal of Computer Vision. 2021;129:1–31.
