# Supplementary material for: AI-Driven Cell Tracking to Enable High-Throughput Drug Screening Targeting Airway Epithelial Repair for Children with Asthma
Source: J Pers Med. 2022 May 17;12(5):809. doi: 10.3390/jpm12050809 (PMC9146422; doi:10.3390/jpm12050809)
Supplement: Supplementary file 1 [file jpm-12-00809-s001.zip › Supplementary Materials/Additional File 4.pdf]

MOT Challenge Submission: EPIC

From: MOTChallenge <info@motchallenge.net>

To: alphonsg@protonmail.com <alphonsg@protonmail.com>

Date: Sunday, September 5th, 2021 at 3:31 PM

Dear Crysis,

Thank you for submitting the results of your method EPIC to MOTChallenge.

Your submission has been evaluated:

Your CTMC-v1 Results

	MOTA	TRA	IDF1	IDP	IDR	TP	FP	FN	Rcll	Prcn	MTR	PTR	MLR	MT	PT	ML	IDSW	FAR	FM
**seq**																			
**3T3-run02**	14.26	37.09	24.63	26.90	22.72	13175	8774	12809	50.70	60.03	13.89	77.78	8.33	5	28	3	695	4.11	1484
**3T3-run04**	45.84	56.56	44.40	44.61	44.19	15164	5207	5401	73.74	74.44	33.33	64.44	2.22	15	29	1	531	2.41	1041
**3T3-run06**	32.48	49.12	43.84	44.19	43.50	9939	4871	5105	66.07	67.11	43.75	40.62	15.62	14	13	5	181	2.90	581
**3T3-run08**	42.23	70.20	62.38	53.98	73.87	3211	1650	341	90.40	66.06	81.82	9.09	9.09	9	1	1	61	1.22	77
**A-10-run02**	17.26	35.28	39.29	43.85	35.58	4469	2835	4533	49.64	61.19	33.33	66.67	0.00	2	4	0	80	1.31	461
**A-10-run04**	-0.08	69.58	66.65	49.98	100.00	1200	1201	0	100.00	49.98	100.00	0.00	0.00	1	0	0	0	1.00	0
**A-10-run06**	56.41	63.84	43.00	41.34	44.80	7075	2039	1336	84.12	77.63	57.14	42.86	0.00	4	3	0	291	1.08	313
**A-549-run02**	48.64	54.81	42.36	43.78	41.03	36396	10985	14157	72.00	76.82	44.44	53.33	2.22	20	24	1	824	4.81	2560
**A-549-run04**	59.04	60.28	46.48	47.42	45.58	25896	5957	7249	78.13	81.30	54.05	43.24	2.70	20	16	1	371	3.17	1373
**APM-run02**	26.51	42.67	25.84	28.14	23.89	9585	4571	7085	57.50	67.71	3.70	92.59	3.70	1	25	1	594	2.21	973

	MOTA	TRA	IDF1	IDP	IDR	TP	FP	FN	Rcll	Prcn	MTR	PTR	MLR	MT	PT	ML	IDSW	FAR	FM
**seq**																			
**APM-run04**	20.08	40.73	28.87	30.98	27.03	7946	4615	6451	55.19	63.26	12.00	76.00	12.00	3	19	3	440	2.18	673
**APM-run06**	-9.92	31.20	18.29	18.73	17.88	10895	12183	13277	45.07	47.21	2.63	76.32	21.05	1	29	8	1109	5.11	1635
**BPAE-run02**	56.45	55.01	53.83	58.70	49.70	35823	6846	14566	71.09	83.96	36.36	58.18	5.45	20	32	3	534	3.18	1820
**BPAE-run04**	44.30	50.47	40.69	44.13	37.75	17254	5290	9095	65.48	76.53	20.83	70.83	8.33	5	17	2	292	2.78	841
**BPAE-run06**	41.10	48.93	34.28	37.25	31.74	16372	5336	9106	64.26	75.42	22.86	62.86	14.29	8	22	5	565	2.52	1326
**CRE-BAG2-run02**	32.87	41.34	29.00	34.58	24.98	51492	18077	44812	53.47	74.02	4.86	85.41	9.73	9	158	18	1759	7.11	5684
**CRE-BAG2-run04**	10.75	46.91	33.41	31.14	36.05	10435	8283	5733	64.54	55.75	22.73	70.45	6.82	10	31	3	414	4.53	838
**CV-1-run02**	-7.05	42.99	51.80	45.66	59.84	549	603	330	62.46	47.66	33.33	33.33	33.33	1	1	1	8	2.06	18
**CV-1-run04**	-84.41	54.92	32.02	22.76	54.02	811	1641	222	78.51	33.08	0.00	100.00	0.00	0	1	0	42	1.56	74
**LLC-MK2-run02b**	86.02	76.29	78.11	77.03	79.22	8454	735	481	94.62	92.00	93.75	6.25	0.00	15	1	0	33	0.88	95
**LLC-MK2-run04**	44.65	55.30	55.02	54.85	55.18	4738	1754	1715	73.42	72.98	50.00	50.00	0.00	3	3	0	103	0.93	211
**LLC-MK2-run06**	19.59	42.82	38.41	39.39	37.47	12059	7796	8809	57.79	60.74	23.53	64.71	11.76	8	22	4	174	4.54	762
**MDBK-run02**	57.33	71.44	78.86	72.35	86.67	867	301	108	88.92	74.23	66.67	33.33	0.00	2	1	0	7	0.52	12
**MDBK-run04**	66.53	60.90	54.31	57.64	51.34	31501	4370	8775	78.21	87.82	58.54	31.71	9.76	24	13	4	334	2.14	1256
**MDBK-run06**	55.72	54.80	49.27	54.29	45.10	18896	3458	8015	70.22	84.53	28.57	63.27	8.16	14	31	4	443	2.01	1376
**MDBK-run08**	55.41	53.11	51.26	58.75	45.45	15648	2436	7727	66.94	86.53	31.43	60.00	8.57	11	21	3	260	1.21	993
**MDBK-run10**	55.35	53.37	45.02	50.76	40.44	33013	5543	15378	68.22	85.62	18.18	74.03	7.79	14	57	6	688	2.31	2776
**MDOK-run02**	-3.63	37.70	33.13	31.95	34.40	3466	3439	2946	54.05	50.20	40.00	60.00	0.00	2	3	0	260	1.72	351
**MDOK-run04**	-92.77	0.00	0.36	0.37	0.36	58	2630	2734	2.08	2.16	0.00	0.00	100.00	0	0	2	18	1.63	26
**MDOK-run06**	31.84	67.80	66.07	56.01	80.54	5097	3200	673	88.34	61.43	75.00	25.00	0.00	3	1	0	60	1.35	88
**MDOK-run08**	6.32	32.68	31.88	35.10	29.19	7230	6016	8694	45.40	54.58	18.75	56.25	25.00	3	9	4	208	3.41	664
**OK-run02**	38.71	48.53	32.79	35.67	30.35	19914	7011	11728	62.94	73.96	20.75	69.81	9.43	11	37	5	654	3.32	1617
**OK-run04**	22.16	47.95	35.02	34.89	35.15	4016	2353	2305	63.53	63.06	11.11	83.33	5.56	2	15	1	262	2.61	423
**OK-run06**	27.09	40.04	31.38	36.63	27.44	15371	6719	14113	52.13	69.58	7.50	77.50	15.00	3	31	6	666	3.51	1863
**PL1Ut-run02**	30.59	41.53	30.55	35.19	26.99	13543	5376	11121	54.91	71.58	11.54	75.00	13.46	6	39	7	622	3.98	1611
**PL1Ut-run04**	32.22	40.19	31.22	37.57	26.70	7464	2587	6676	52.79	74.26	10.53	84.21	5.26	2	16	1	321	1.25	698

	MOTA	TRA	IDF1	IDP	IDR	TP	FP	FN	Rcll	Prcn	MTR	PTR	MLR	MT	PT	ML	IDSW	FAR	FM
**seq**																			
**RK-13-run02**	66.52	61.23	60.81	64.06	57.87	12643	1794	3338	79.11	87.57	55.00	45.00	0.00	11	9	0	219	1.71	703
**U2O-S-run02**	52.10	50.97	34.05	38.82	30.32	90924	17112	47389	65.74	84.16	25.68	66.22	8.11	19	49	6	1753	3.86	7100
**U2O-S-run04**	47.64	51.98	39.78	42.68	37.25	29257	8277	13747	68.03	77.95	19.05	68.25	12.70	12	43	8	493	3.89	1939
**OVERALL**	41.22	49.76	39.17	42.39	36.40	611846	203871	338080	64.41	75.01	24.38	66.51	9.11	313	854	117	16369	2.83	46336

Kind Regards,

The MOTChallenge Team

https://motchallenge.net
