# Supplementary material for: AI-Driven Cell Tracking to Enable High-Throughput Drug Screening Targeting Airway Epithelial Repair for Children with Asthma
Source: J Pers Med. 2022 May 17;12(5):809. doi: 10.3390/jpm12050809 (PMC9146422; doi:10.3390/jpm12050809)
Supplement: Supplementary file 1 [file jpm-12-00809-s001.zip › Supplementary Materials/Additional File 5.html]

 
 
  
 

 report_auto_tracks   


 


 

     

 

 
     
     
     
      
 

  
 Low-Resolution and Low-Framerate AI-Enabled Cell Tracking using Bounding Boxes for High-Throughput Drug Screening ¶   Autogenerated Report: EPIC Cell Migration Analysis ¶  
 
  

  

  

 
  
 Image Series ¶  
 
  

 

 

 

 
 Loaded 22 images.
 
 
 

 

 

  

 

 

 

 
 Image 1: NuLi-1_30 mins_D7_1_2014y10m16d_13h30m.jpg
Image 2: NuLi-1_30 mins_D7_1_2014y10m16d_14h00m.jpg
Image 3: NuLi-1_30 mins_D7_1_2014y10m16d_14h30m.jpg
Image 4: NuLi-1_30 mins_D7_1_2014y10m16d_15h00m.jpg
Image 5: NuLi-1_30 mins_D7_1_2014y10m16d_15h30m.jpg
Image 6: NuLi-1_30 mins_D7_1_2014y10m16d_16h00m.jpg
Image 7: NuLi-1_30 mins_D7_1_2014y10m16d_16h30m.jpg
Image 8: NuLi-1_30 mins_D7_1_2014y10m16d_17h00m.jpg
Image 9: NuLi-1_30 mins_D7_1_2014y10m16d_17h30m.jpg
Image 10: NuLi-1_30 mins_D7_1_2014y10m16d_18h00m.jpg
Image 11: NuLi-1_30 mins_D7_1_2014y10m16d_18h30m.jpg
Image 12: NuLi-1_30 mins_D7_1_2014y10m16d_19h00m.jpg
Image 13: NuLi-1_30 mins_D7_1_2014y10m16d_19h30m.jpg
Image 14: NuLi-1_30 mins_D7_1_2014y10m16d_20h00m.jpg
Image 15: NuLi-1_30 mins_D7_1_2014y10m16d_20h30m.jpg
Image 16: NuLi-1_30 mins_D7_1_2014y10m16d_21h00m.jpg
Image 17: NuLi-1_30 mins_D7_1_2014y10m16d_21h30m.jpg
Image 18: NuLi-1_30 mins_D7_1_2014y10m16d_22h00m.jpg
Image 19: NuLi-1_30 mins_D7_1_2014y10m16d_22h30m.jpg
Image 20: NuLi-1_30 mins_D7_1_2014y10m16d_23h00m.jpg
Image 21: NuLi-1_30 mins_D7_1_2014y10m16d_23h30m.jpg
Image 22: NuLi-1_30 mins_D7_1_2014y10m17d_00h00m.jpg
 
 
 

 

 

  

 

 

 


 
 
  
 Your browser does not support the video tag.
  
 

 

 

 

 
  
 Cell Detections ¶  
 
  

  

 

 

 

 
 Loaded 69595 detections.
 
 
 

 

 

  

 

 

 

 
 Image 1 contains 3134 detections
Image 2 contains 3194 detections
Image 3 contains 3095 detections
Image 4 contains 3137 detections
Image 5 contains 3102 detections
Image 6 contains 3079 detections
Image 7 contains 3138 detections
Image 8 contains 3107 detections
Image 9 contains 3109 detections
Image 10 contains 3115 detections
Image 11 contains 3132 detections
Image 12 contains 3097 detections
Image 13 contains 3131 detections
Image 14 contains 3164 detections
Image 15 contains 3142 detections
Image 16 contains 3130 detections
Image 17 contains 3187 detections
Image 18 contains 3236 detections
Image 19 contains 3289 detections
Image 20 contains 3253 detections
Image 21 contains 3278 detections
Image 22 contains 3346 detections

Average number of detections per image: 3163
 
 
 

 

 

  

 

 

 


 
 
  
 Your browser does not support the video tag.
  
 

 

 

 

 
  
 Leading Edges ¶  
 
  

 

 

 

 
 Detected top and bottom leading edges, in first image of series, at y-positions 471 and 1161 (px.) respectively.
 
 
 

 

 

  

 

 

 


 
 <matplotlib.image.AxesImage at 0x1a1edbf38e0> 
 

 

 


 
 
 

 

 

 

 
  
 Cell Tracks ¶  
 
  

 

 

 

 
 Loaded 9240 tracks.
 
 
 

 

 

  

 

 

 

 
 Average length of tracks: 8 frames
Average starting frame of tracks: frame 9
Average ending frame of tracks: frame 15
 
 
 

 

 

  

 

 

 


 
 
  
 Your browser does not support the video tag.
  
 

 

 

 

 
  
 Sampled Cell Tracks ¶  
 
  

  

  

 

 

 

 
 Sampled Automated Tracks
Number of tracks longer than 21 frames: 539
Number of sampled cells: 20
 
 
 

 

 

 
  
 Cell Migration Metrics ¶  
 
  

  

 

 

 

 
 Metric: Euclidean Distance
Automated average: 143.12 Micrometres (um)

Metric: Accumulated Distance
Automated average: 220.8 Micrometres (um)

Metric: Velocity
Automated average: 13.63 Micrometres/Hour (um/hr)

Metric: Directionality
Automated average: 0.64 Arbitrary Units (AU)

Metric: Y - Forward Motion Index
Automated average: 0.85 Arbitrary Units (AU)

Metric: End Point Angle
Automated average: 91.73 Degrees (Deg)

 
 
 

 

 

  

 

 

 


 
 
 

 

 

 

  

 

 

 


 
 
 

 

 

 

  

 

 

 


 
 
 

 

 


 
 
 

 

 

 

 
 


 


 
